# Microbial signature of intestine in children with allergic rhinitis

**DOI:** 10.3389/fmicb.2023.1208816

**Published:** 2023-07-25

**Authors:** Panpan Zhang, Xuehua Zhou, Hong Tan, Fangfang Jian, Zenghui Jing, Huajie Wu, Yao Zhang, Jianfeng Luo, Juan Zhang, Xin Sun

**Affiliations:** Department of Pediatrics, Xijing Hospital, The Fourth Military Medical University, Xi’an, Shaanxi, China

**Keywords:** allergic rhinitis, children, 16S rRNA gene sequencing, gut, microbiota

## Abstract

**Introduction:**

Previous studies have found that unique patterns of gut microbial colonization in infancy associated with the development of allergic diseases. However, there is no research on the gut microbiota characteristics of AR children in Chinese Mainland.

**Objective:**

To investigate the changes of gut microbial of AR children in Chinese Mainland and evaluate the correlation between gut microbial and clinical indexes.

**Methods:**

In this clinical study, fecal samples from 24 AR children and 25 healthy control children (HCs) were comparative via next generation sequencing of the V3-V4 regions of the 16S rRNA gene. Analyzed the relationship between clinical features and gut microbial using Spearman correlation.

**Results:**

Compared to HCs, AR children showed significant decreases in Shannon index and significant increases in Simpson index at both the family and genera levels (all *p* < 0.05). In terms of bacterial composition, at the phylum level, AR children had higher abundance of *Bacteroidetes* than that in the HCs group (*p* < 0.05) and were significantly positively correlated with TNSS (*p* < 0.05). At the family level, AR children had higher abundance of *Prevotellaceae* and *Enterobacteriaceae* higher than that in the HCs group (all *p* < 0.05) and had a significantly positive correlation with TNSS, eosinophils (EOS) and total immunoglobulin E (tIgE) (all *p* < 0.05). At the genus level, reduced abundance of *Agathobacter, Parasutterella, Roseburia* and *Subdoligranulum* were also observed in the AR cohorts compared to HCs (all *p* < 0.05) and significantly negatively associated with TNSS, EOS, tIgE, QOL, and FeNO (all *p* < 0.05).

**Conclusion:**

AR children in Chinese Mainland were characterized by reduced microbial diversity and distinguished microbial characteristics in comparison with HCs. The observations of this study offer proof that distinctive gut microbiota profiles were present in AR children and necessitate further investigation in the form of mechanistic studies.

## 1. Introduction

Allergic rhinitis (AR) is a common allergic disease characterized by paroxysmal sneezing, nasal itching, nasal congestion, and runny nose caused by IgE-mediated immune responses to inhaled allergens (Ciprandi et al., 2015). The immune response involves inflammation of the mucosa, which is driven by the activation of type 2 helper T (Th2) cells (Bousquet J. et al., 2008). AR affects 10 to 40% of the world’s population, with a higher prevalence as high as 50% in some countries (Bousquet P. J. et al., 2008; Brożek et al., 2017). The prevalence of AR among Chinese children has been reported to be 15.79%, and it continues to increase (Hu et al., 2017). AR contributes to inefficiency at school, sleep problems, and reduces children’s chances of participating in outdoor activities (Devillier et al., 2016). Regretfully, the exact etiology of AR and its underlying biological mechanism are still unclear (Emeryk et al., 2019). A growing number of studies in recent years have suggested that imbalances in microecology are related to the occurrence of allergic disease (Zimmermann et al., 2019; Barcik et al., 2020; Shah and Bunyavanich, 2021). Microecology dysbiosis, especially in genetically susceptible hosts, may be a cofactor in the development of allergic diseases due to its role in undermining immune balance, including the proportion of effector T cells and regulatory T (Treg) cells (Salem et al., 2018).

In the 1980s, Strachan proposed the “hygiene hypothesis”, which suggests that children living in rural areas, having close contact with animals and drinking unpasteurized cow’s milk had a lower incidence of developing allergic diseases in the future (Huang et al., 2017). In 2015, the “microbiome hypothesis” was proposed, which suggested that the disruption of gut microbiome-mediated immune tolerance due to antibiotic use, infection, and other factors could eventually promote the development of the immune system towards allergy. The symbiosis between host and microbiome plays an essential role in maintaining immune homeostasis and human health, thus, changes in the gut microbiota are believed to be an important factor in the development of allergic disease (Mccoy and Köller, 2015).

Gut microbiota is in dynamic equilibrium and can regulate itself according to the change of environment. Children’s gut microbiota is less resistant and stable than adults and is sensitive to external factors such as the use of antibiotic and glucocorticoids (Fang et al., 2021). Glucocorticoids have remarkable local anti-inflammatory and anti-allergic effects and are the most effective drugs to treat allergic diseases in children, it could directly or indirectly affect gut microbial (Zhang et al., 2021; Liu et al., 2022). At the same time, antibiotics are widely used clinically, they could upset the balance of gut microbiota, leading to decline in microbial diversity and the overgrowth of bacteria such as *Clostridium difficile* (Ianiro et al., 2020).

With the development of the amplification and sequencing of the 16S rRNA gene, we can obtain the distribution map of microbial species presented in different allergic diseases (Su et al., 2021; Yuan et al., 2022). Currently, clinical studies have demonstrated alterations in the composition and function of the gut microbiome in adults and children with AR (Watts et al., 2021; Zhou et al., 2021). However, there is no study on the gut microbiome of children with AR in Chinese Mainland.

Therefore, in order to investigate the alters of children with AR in Chinese Mainland and evaluate the relationship between the gut microbiota and clinical indicators such as Total Nasal Symptom Score (TNSS), Visual Analog Scale (VAS), Quality of Life Score (QOL), Fractional exhaled nitric oxide (FeNO) value etc. We conducted this study, which will contribute to a better understanding of the underlying biological mechanisms of AR, and help to develop more effective prevention and treatment strategies for AR in children.

## 2. Materials and methods

### 2.1. Study design

This prospective and cross-sectional observational study was conducted with ethics approval from the Ethics Committee of Xijing Hospital at the Fourth Military Medical University in Xi’an, China (KY20222169-F-1), and was registered with the Chinese Clinical Trial Registry (ChiCTR2200065166). The study enrolled subjects from the Department of Pediatrics at Xijing Hospital during the period of July to December 2022. All parents or guardians of the participants provided written consent, and the study was conducted in accordance with the ethical principles outlined in the Declaration of Helsinki.

### 2.2. Study population

This is a description of the diagnostic and inclusion/exclusion criteria used in a clinical study of participants. AR diagnosis relied on the ARIA guidelines, which includes both seasonal and perennial AR (Cheng et al., 2018). After 1 month of treatment, a follow-up assessment was conducted to confirm the diagnosis of AR.

The inclusion criteria of AR group required children to have had a history of allergies in the past year, the presence of allergen-specific IgE antibody (sIgE) test ≥0.35 IU/mL and/or positive skin prick tests (SPT), aged 3-14 years, stools that appeared snake-shaped or banana-shaped. The inclusion criteria of HCs group required children without allergic history, aged 3–14 years, and stools that appeared snake-shaped or banana-shaped. The exclusion criteria of both groups were numerous and included as follows: using immunomodulatory medications in the past 12 months; using probiotics, prebiotics and synbiotics in the past 2 months; using antibiotics, local or systemic corticosteroids, antihistamines in the past 2 weeks; using laxatives or antidiarrheals in the past 1 week, having constipation, diarrhea or respiratory tract infection in the past week; having congenital heart disease, tuberculosis or tuberculosis contact history, mental illness history, autoimmune disease, gastrointestinal disease, and allergic asthma (Zhu et al., 2020; Zhou et al., 2021). The study participants were selected from the same geographic region and had similar dietary habits.

### 2.3. Stool sample collection

Before the start of the treatment, the parents of the study participants were provided with a fecal sample collection kit and dry ice, and were instructed on how to collect the stool sample. The collection instructions emphasized the importance of avoiding contamination of the samples with urine or water, and the samples were frozen immediately, and carefully transported to our laboratory using dry ice within 24 h. The samples were stored at a temperature of −80°C until they were processed (Chiu et al., 2019).

### 2.4. Total nasal symptom score

Four nasal symptoms (sneezing, nasal itching, rhinorrhea, nasal obstruction) were assessed using a four-point scale. The scale ranged from 0 to 3, where 0 indicated the absence of symptoms, 1 indicated mild symptoms, 2 indicated moderate symptoms, and 3 indicated severe symptomss (Gevaert et al., 2022).

### 2.5. Visual analog scale

At the initial consultation, the children were requested to indicate the intensity of their allergic rhinitis symptoms using a scale ranging from 0 to 10. A score of 0 meant that the child was asymptomatic, while a score of 10 indicated that the symptoms were most severe (Eifan et al., 2010).

### 2.6. Quality of life score

Quality of life scores of AR children are shown in Supplementary Table S1. During the initial consultation, the child or their guardian was required to mark the applicable space in the table that reflected the impact of the disease on the child’s life in the past 1–2 weeks. The quality of life scores in the supplementary table ranged from low to high, with higher scores indicating a worse quality of life (Okubo et al., 2020).

### 2.7. Microbiome analysis

To analyze the diversity and composition of the microbial community, DNA was extracted from fecal samples, the concentration and purity were measured using the NanoDrop One (Thermo Fisher Scientific, MA, United States). 16S rRNA genes of V3-V4 regions were amplified used specific primer 338F (5’-ACTCCTACGGGAGGCA GCAG-3′) and 806R (5’-GGACTACHVGGGTWTCTAAT-3′) with 12 bp barcode. Primers were synthesized by Invitrogen (Invitrogen, Carlsbad, CA, United States). Sequencing libraries were generated using NEBNext^®^ Ultra^™^ II DNA Library Prep Kit for Illumina^®^ (New England Biolabs, MA, United States) following manufacturer’s recommendations and index codes were added. The library quality was assessed on the Qubit@ 2.0 Fluorometer (Thermo Fisher Scientific, MA, United States). At last, the library was sequenced on an Illumina Nova6000 platform and 250 bp paired-end reads were generated (Guangdong Magigene Biotechnology Co., Ltd. Guangzhou, China).

Fastp (version 0.14.1, https://github.com/OpenGene/fastp) was used to control the quality of the Raw Data by sliding window, the primers were removed by using cutadapt software (https://github.com/marcelm/cutadapt/) according to the primer information at the beginning and end of the sequence to obtain the paired-end Clean Reads. Paired-end clean reads were merged using usearch-fastq_mergepairs (V10, http://www.drive5.com/usearch/), Fastp (version 0.14.1, https://github.com/OpenGene/fastp) was used to control the quality of the Raw Data by sliding window(-W 4 -M 20) to obtain the paired-end Clean Tags.

Operational taxonomic units (OTUs) were generated using the UPARSE default clustering approach. Taxonomic information was annotated using the Silva database and the usearch -sintax tool. Alpha diversity was analyzed using three indicators: chao1 index, Shannon index, and Simpson index. All this indices in our samples were calculated with usearch-alpha_div (V10, http://www.drive5.com/usearch/). The differences between groups were analyzed by alpha diversity index using R software. If there were only two groups, student’s *t*-test or Wilcox rank sum test was selected. Beta diversity was analyzed using the Bray-Curtis algorithm. PCoA was displayed by vegan package in R software. To identify biomarkers for each group, linear discriminant analysis (LDA) and linear discriminant analysis effect size (LEfSe) analysis were performed, setting LDA score ≥ 3.5 and obtained the biomarkers in different groups. All analyses were completed on the Magichand Cloud platform.

### 2.8. Statistical analyses

SPSS software (v.26.0) was used for the analysis of the baseline characteristics data. Continuous variables were reported as Mean ± SD, student *t*-test or Wilcoxon signed-rank test method was used for inter-group comparison; count data were reported as *n* (%), χ^2^ test or Fisher’s exact probabilities test was used for inter-group comparison. Correlation analysis between the clinical index and gut bacteria using Spearman’s correlation coefficient. *p* value < 0.05 was considered statistically significant.

## 3. Results

### 3.1. Characteristics of all participants

A total of 49 participants were enrolled in this study, including 24 children with AR and 25 HCs. Table 1 presents the clinical demographics of the study. Basic natural characteristics such as age, gender, and BMI (Body Mass Index), birth history including term birth and cesarean delivery, feeding history including breastfeeding for at least 3 months, and living environment such as the presence of siblings, exposure to passive smoking, and exposure to pets did not show significant differences between the two groups (all *p* > 0.05). However, the number of maternal allergy was significantly higher in the AR group compared to the HCs (*p* < 0.05). All AR children had positive sIgE results, 83.3% of the AR children had seasonal allergies, and 87.5% are in acute attack.

**Table 1:** Clinical characteristics of participants.

	HCs group	AR group
Subject (*n*)	25	24
Age (years, Mean ± SD)	6.72 ± 2.56	7.42 ± 2.89
BMI(kg/m^2^, Mean ± SD)	17.36 ± 4.27	15.98 ± 3.58
Male *n* (%)	15 (60.0)	15 (62.5)
Term birth *n* (%)	24 (96.0)	22 (91.7)
Cesarean *n* (%)	11 (44.0)	13 (54.2)
Breastfeeding for at least 3 months *n* (%)	19 (76.0)	17 (70.8)
Maternal allergy *n* (%)	2 (8.0)	8 (33.3)*
Siblings *n* (%)	14 (56.0)	11 (45.8)
Passive smoking *n* (%)	13 (52.0)	6 (25.0)
Pets *n* (%)	5 (20.0)	4 (16.7)
Positive of sIgE *n* (%)	0 (0)	24 (100)*
Seasonal allergies *n* (%)	0 (0)	20 (83.3)*
Acute attack *n* (%)	0 (0)	21 (87.5)*
TNSS (Mean ± SD)	1.58 ± 1.17	5.96 ± 1.73*
Sneezing	0.58 ± 0.50	1.75 ± 0.90*
Rhinorrhea	0.50 ± 0.59	1.29 ± 0.55*
Nasal itching	0.29 ± 0.46	1.21 ± 0.41*
Nasal obstruction	0.21 ± 0.41	1.71 ± 0.62*
VAS (Mean ± SD)	0.64 ± 0.56	4.85 ± 2.22*
QOL (Mean ± SD)	0.88 ± 0.67	3.21 ± 2.62*
tIgE (IU/ml, Mean ± SD)	<17.10	206.52 ± 154.13*
FeNO (ppb, Mean ± SD)	9.84 ± 3.40	18.91 ± 13.56*

AR, allergic rhinitis group; HCs, healthy control group; TNSS, total nasal symptom Score; VAS, visual analog scale; QOL, quality of life score; tIgE, total immunoglobulin E; FeNO, fractional exhaled nitric oxide *there is a statistical difference between the two groups, *p* < 0.05.

### 3.2. Gut microbiota of AR children versus HCs

**Figure 1:**
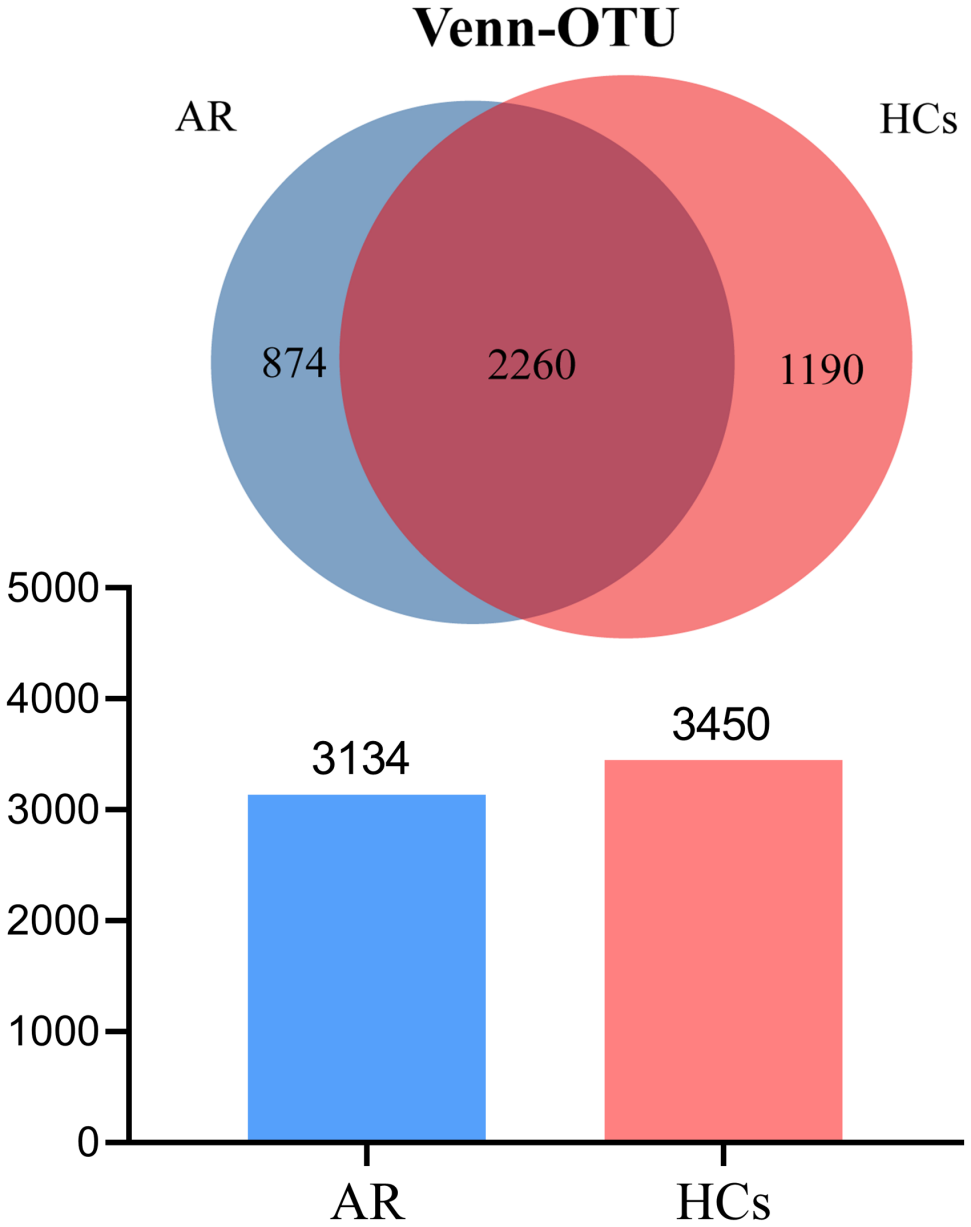
Venn plot. The total number of OTUs observed in the AR group was lower than that in the HCs group. AR, allergic rhinitis group; HCs, healthy control group.

The 16S rRNA gene sequencing was performed on the Illumina Nova 6,000 platform. A total of 4,573,805 high-quality sequences were acquired from 49 samples, after quality control filtering and minimum sample OTU normalization, averaging 61,446 effective sequences per sample (Supplementary Table S2). The Venn diagram showed that the number of OTUs in the AR group was 3,134, while that in the HCs group was 3,450. There were 2,260 OTUs shared by both groups, 874 OTUs unique to the AR group, and 1,190 OTUs unique to the HCs group. The total number of observed OTUs in the AR group was lower than that in the HCs group (Figure 1).

#### 3.2.1. Alpha-diversity

**Figure 2:**
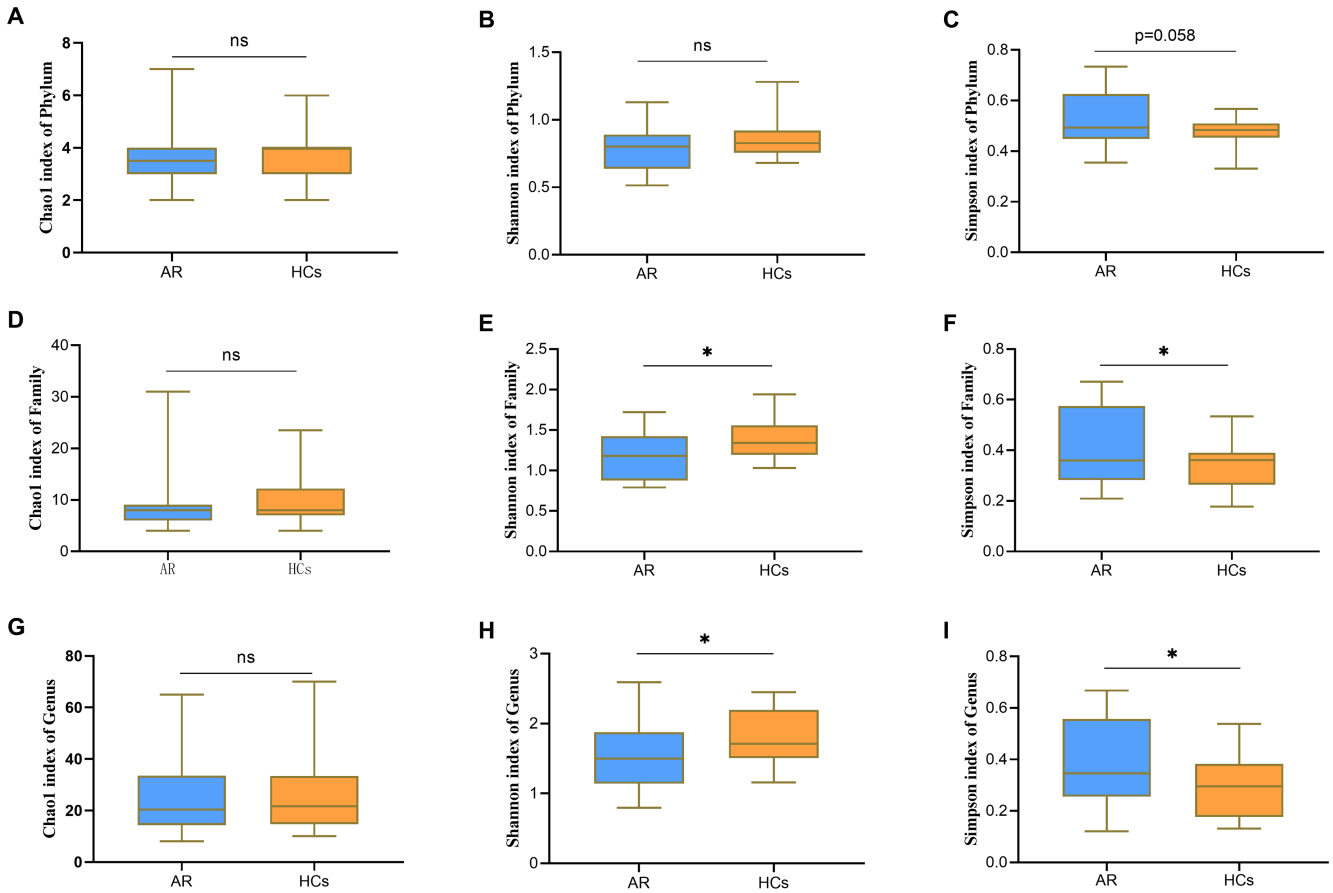
Alpha-Diversity analysis chart. The Alpha-Diversity indexes comparing including Chao1, Shannon and Simpson. **(A)** Chao1 index at the phylum level; **(B)** Shannon index at the phylum level; **(C)** Simpson index at the phylum level; **(D)** Chao1 index at the family level; **(E)** Shannon index at the family level; **(F)** Simpson index at the family level; **(G)** Chao1 index at the genus level; **(H)** Shannon index at the genus level; and **(I)** Simpson index at the genus level. AR = allergic rhinitis group; HCs, healthy control group. ns, no statistical difference between the two groups; *there is a statistical difference between the two groups, *p* < 0.05.

At the phylum level, there was no statistically significant difference between the two groups for both Chao1 index and Shannon index (all *p* > 0.05, Figures 2A,B), but there was a statistically significant difference for the Simpson index (*p* = 0.058, Figure 2C). At the family level, there was no statistically significant difference between the two groups for the Chao1 index (*p* > 0.05, Figure 2D), but there was a statistically significant difference for both the Shannon and Simpson indices (all *p* < 0.05, Figures 2E,F). At the genus level, there was no statistically significant difference between the two groups for the Chao1 index (*p* > 0.05, Figure 2G), but there was a statistically significant difference for both the Shannon and Simpson indices (all *p* < 0.05, Figures 2H,I). The outcomes mentioned above suggest that there is no variation in species richness between the two categories at all levels. However, a significant divergence in evenness exists, and it becomes more explicit as the taxonomic hierarchy becomes more detailed (Supplementary Table S3).

#### 3.2.2. Beta-diversity

**Figure 3:**
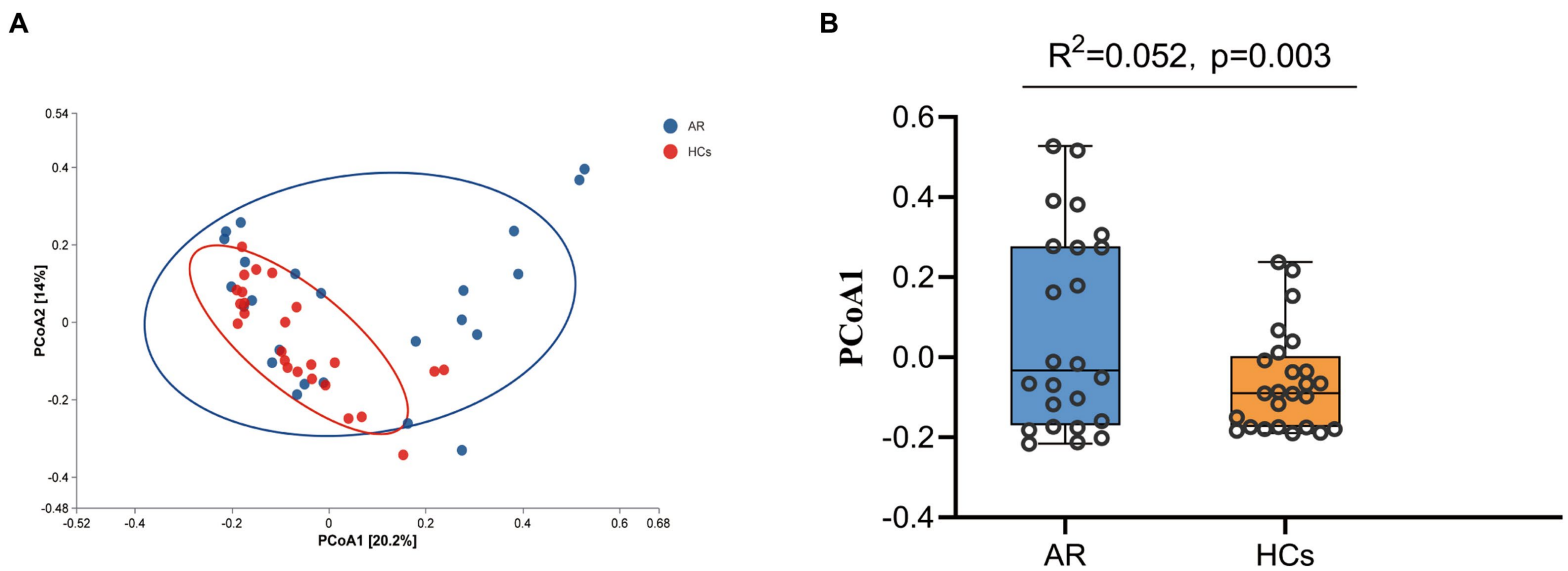
Beta-Diversity Analysis Chart. The Beta-Diversity including PcoA and Adonis analysis. **(A)** PCoA analysis results based on Bray curtis and **(B)** PCoA1 difference based on Adonis analysis. AR, allergic rhinitis group; HCs, healthy control group.

Figure 3A shows the PCoA based on Bray–Curtis dissimilarity analysis. The PCoA1 from Adonis analysis showed that *R*^2^ = 0.052, *p* = 0.003, indicating a significant difference between the two groups (Figure 3B).

#### 3.2.3. Microbial composition classification

The sequence information of the three taxonomic levels of phylum, family, and genus in the OTU table was counted to calculate the relative abundance of microbial communities at each taxonomic level. The taxa with relative abundance greater than 1% were selected to draw a relative abundance diagram. Student’s *t*-test or Wilcoxon rank sum test were used to compare the differences in microbial communities (Supplementary Table S4).

**Figure 4:**
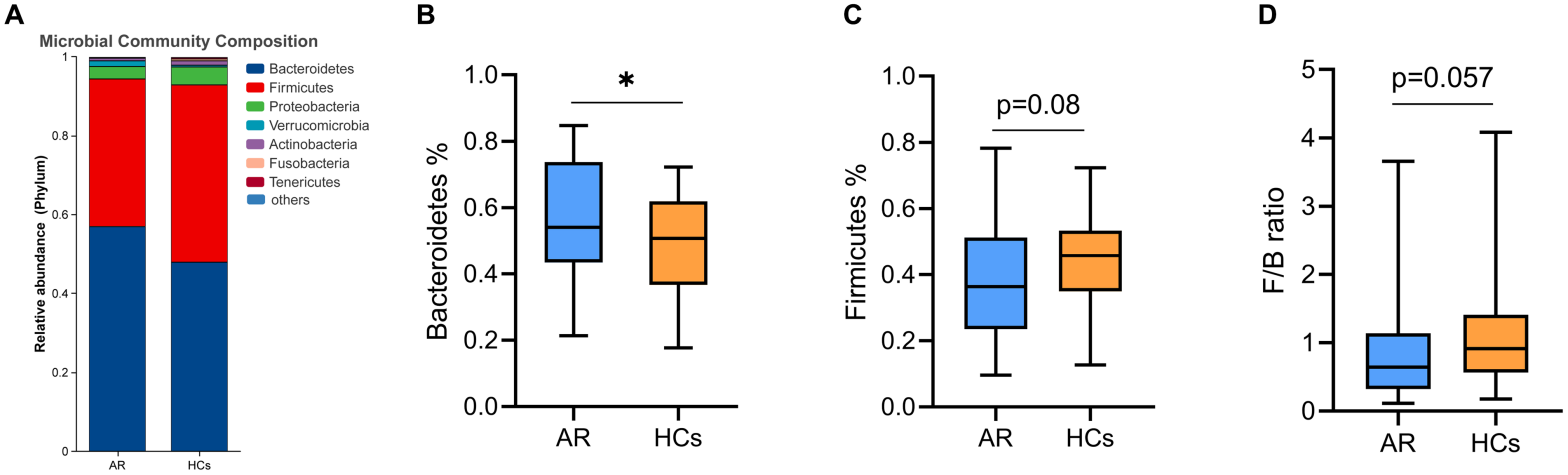
The difference of gut microbiota at phylum level. **(A)** Community composition at the phylum level; **(B)** Inter-group differences of Bacteroidetes in phylum; **(C)** Inter-group differences of Firmicutes in phylum; and **(D)** Inter-group differences in the ratio of Firmicutes to Bacteroidetes. AR, allergic rhinitis group; HCs, healthy control group. *there is a statistical difference between the two groups, *p* < 0.05.

At the phylum level, *Bacteroidetes*, *Firmicutes, Proteobacteria, Verrucomicrobia*, and *Actinobacteria* were the dominant phyla in the two groups (Figure 4A). The relative abundance of *Bacteroidetes* in the AR group was significantly higher than that in the HCs group (*p* < 0.05, Figure 4B), while the relative abundance of *Firmicutes* in the AR group was lower than in the HCs group (*p* = 0.08, Figure 4C). In addition, the ratio of *Firmicutes* to *Bacteroidetes* also differed between the two groups (*p* = 0.057, Figure 4D).

**Figure 5:**
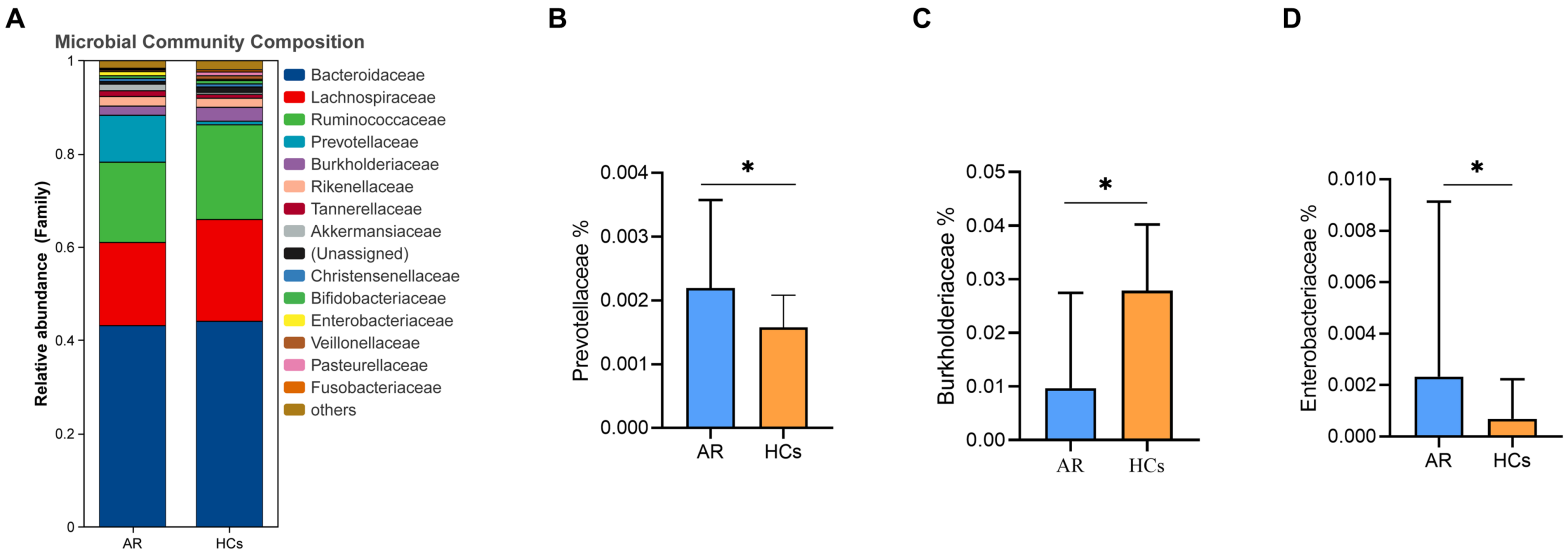
The difference of gut microbiota at family level. **(A)** Community composition at the family level; **(B)** Inter-group differences of Prevotellaceae in family; **(C)** Inter-group differences of Burkholderiaceae in family; **(D)** Inter-group differences of Enterobacteriaceae in family. AR, allergic rhinitis group; HCs, healthy control group. *there is a statistical difference between the two groups, *p* < 0.05.

At the family level, *Bacteroidaceae, Lachnospiraceae, Ruminococcaceae*, and *Prevotellaceae* were the dominant families in the two groups (Figure 5A). The relative abundance of *Prevotellaceae* and *Enterobacteriaceae* in the AR group was significantly higher than that in the HCs group (all *p* < 0.05, Figures 5B,D). While the relative abundance of *Burkholderiaceae* in the AR group was significantly lower than HCs (*p* < 0.05, Figure 5C).

**Figure 6:**
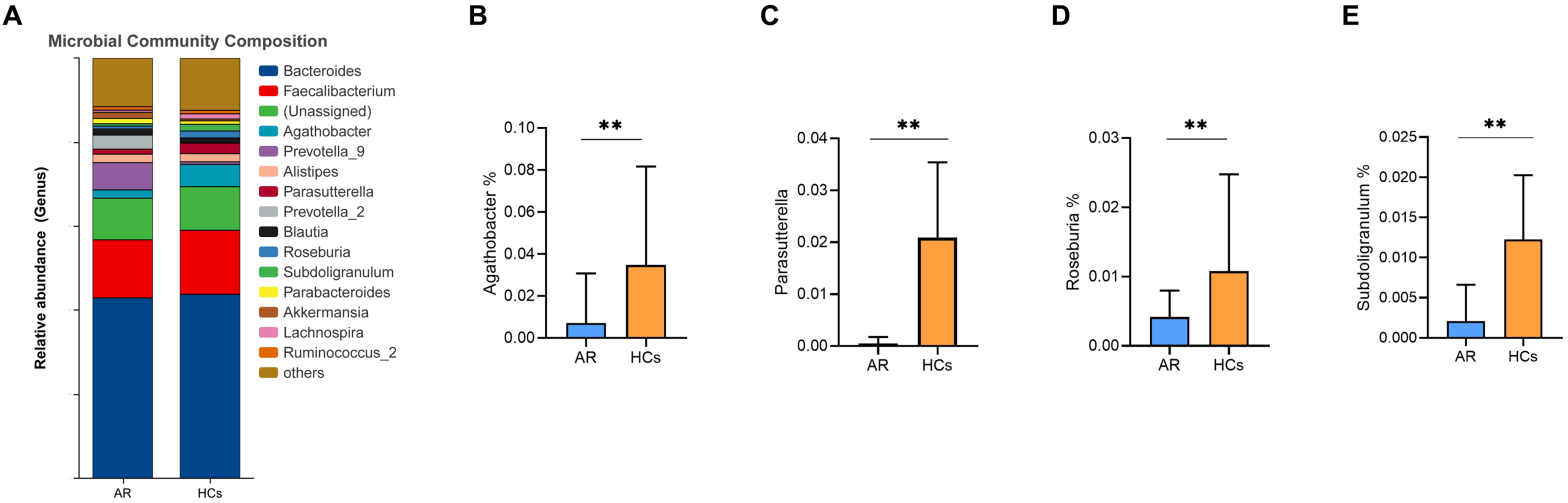
The difference of gut microbiota at genus level. **(A)** Community composition at the genus level; **(B)** Inter-group differences of Agathobacter in genus; **(C)** Inter-group differences of Parasutterella in genus; **(D)** Inter-group differences of Roseburia in genus; **(E)** Inter-group differences of Subdoligranulum in genus. AR, allergic rhinitis group; HCs, healthy control group. **there is a statistical difference between the two groups, *p* < 0.01.

At the genus level, *Bacteroides, Faecalibacterium, Agathobacter, Prevotella_9, Alistipes, Parasutterella, Prevotella_2, Blautia, Roseburia* and *Subdoligranulum* were the dominant genus in the two groups (Figure 6A). Among them, the relative abundance of *Agathobacter, Parasutterella, Roseburia* and *Subdoligranulum* in the AR group was lower than that in the HCs group (all *p* < 0.05, Figures 6B–E).

#### 3.2.4. LEfSe analyze

**Figure 7:**
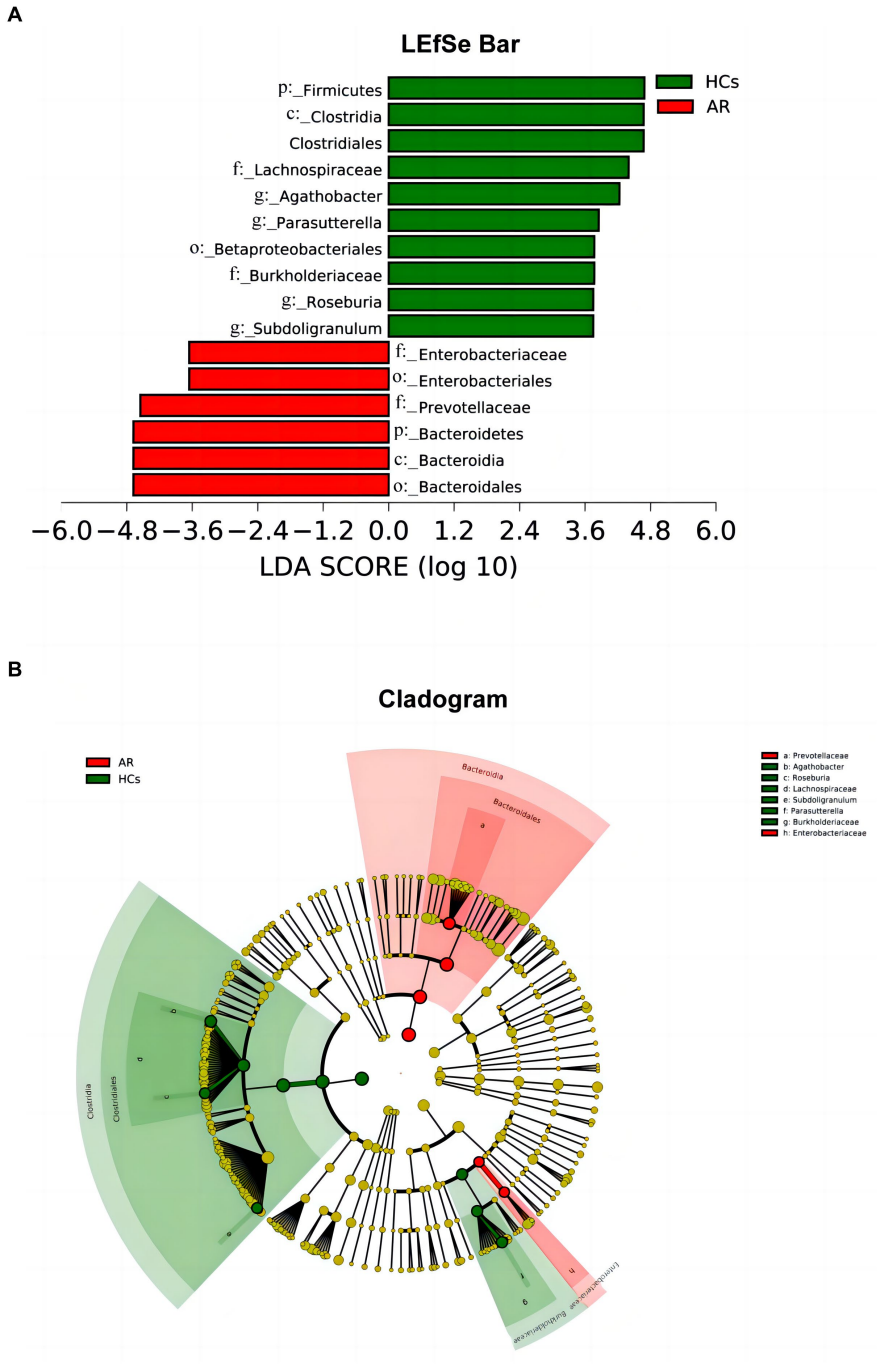
LEfSe chart. LEfSe analysis showed differential taxa with LDA scores greater than 3.5 between two groups **(A)** linear discriminant analysis (LDA) distribution histogram (LDA > 3.5, *p* < 0.05) and **(B)** Cladogram. AR, allergic rhinitis group; HCs, healthy control group.

LEfSe analysis is one of the tools used for identifying and interpreting biological markers in high-dimensional data. It can also be applied to compare groups to identify species (or biomarkers) with significant differences in abundance between those groups. Figures 7A,B shows the differential bacterial taxa with LDA scores above 3.5. The relative abundance of *Firmicutes, Lachnospiraceae, Agathobacter, Parasutterella, Roseburia*, and *Subdoligranulum* was higher in the HCs group than that in the AR group. However, the relative abundance of *Bacteroidetes, Prevotellaceae*, and *Enterobacteriaceae* was higher in the AR group compared to the HCs.

### 3.3. Association of gut microbiota related to clinical indicators

**Figure 8:**
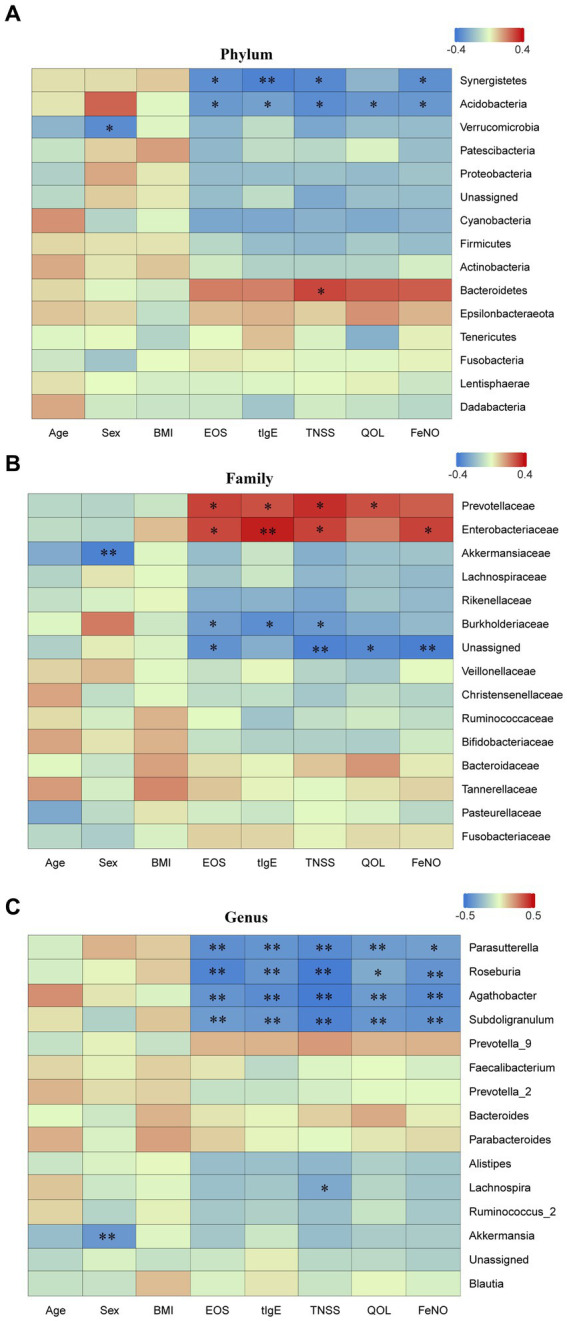
Spearman correlation analysis. Spearman correlation analysis was performed to examine the correlation between different taxa at various levels and clinical indicators. **(A)** phylum level; **(B)** family level; and **(C)** genus level. EOS, eosinophils; tIgE, total immunoglobulin E; TNSS, total nasal symptom score; QOL, quality of life score; FeNO, fractional exhaled nitric oxide. Red represents positive correlation, while blue represents negative correlation. AR, allergic rhinitis group; HCs, healthy control group. *there is a statistical difference between the two groups, *p* < 0.05. **there is a statistical difference between the two groups, *p* < 0.01.

Spearman correlation analysis was performed to investigate the correlation between different bacterial taxa and clinical indicators at the different levels. At the phylum level, *Bacteroidetes* was positively correlated with TNSS (*p* < 0.05), while *Synergistetes* and *Acidobacteria* were negatively correlated with EOS, tIgE, TNSS, and FeNO (all *p* < 0.05) (Figure 8A). At the family level, *Enterobacteriaceae* was positively correlated with EOS, tIgE, TNSS, and FeNO; *Prevotellaceae* was positively correlated with EOS, tIgE, TNSS, and QOL (all *p* < 0.05), while *Burkholderiaceae* was negatively correlated with EOS, tIgE and TNSS (all *p* < 0.05) (Figure 8B). At the genus level, *Parasutterella, Roseburia, Agathobacter*, and *Subdoligranulum* were negatively correlated with EOS, tIgE, TNSS, QOL, and FeNO (all *p* < 0.05) (Figure 8C).

## 4. Discussion

An increasing amount of research suggests that gut microbiota dysbiosis was closely linked to the incidence and development of allergic diseases (Arrieta et al., 2015; Fujimura et al., 2016; Khadka et al., 2021). However, only one past study investigated the link between gut microbiota and childhood AR, but this study is limited to the Taiwan region (Chiu et al., 2019). Considering that different regional environments may have an impact on microecology. The aims of this study was to investigate the alters of children with AR in Chinese Mainland and evaluate the relationship between the gut microbiota and clinical indicators.

In this research, we observed that the alpha diversity in the gut microbiota was decreased, beta diversity and gut microbiota structure were significantly changed in AR children compared to HCs. The conclusions of our study showed a more significant statistical difference compared to the previous ones (Chiu et al., 2019). This study mainly found that the occurrence of AR in children is associated with changes in gut microbiota diversity. As compared to HCs, the Shannon index in AR children was significantly reduced, particularly at the family and genus levels. Similar trends were observed with the Simpson index and OTU count. Reduced gut microbiota diversity weakens their ability to resist external interferences, and it is believed to be an important indicator of whether the gut microbiota is stable or not (Bäckhed et al., 2012). Other studies also shown that people with allergic diseases have lower gut microbiota diversity than healthy ones. Hua et al. analyzed 16S rRNA gene data obtained from US public database and found that the increased incidence of allergic diseases in adults was positively correlated with the reduction in gut microbiota diversity (Melli et al., 2016). Meanwhile, Bisgaard et al. reported that the decrease in gut microbiota diversity at 1 month and 12 months after birth increased the risk of AR in children in the first 6 years of life (Bisgaard et al., 2011). All of the above studies suggest that a decrease of gut microbiota diversity may influence the development of allergic diseases.

This study also found that the gut microbiota composition of children with AR significantly differs from HCs at various levels. At the phylum level, there is a certain difference in the proportion of *Bacteroidetes* and *Firmicutes* between the two groups, with a higher relative abundance of *Bacteroidetes* in AR children. Similar findings of *Bacteroidetes* abundance in fecal samples of allergy sufferers have been reported in other studies. Data analysis from the American Gut Project revealed that the phylum *Bacteroidetes* was more abundant in adults with nut and pollen allergies than in healthy adults (Melli et al., 2016). A study from Japan also showed that infants with high abundance of *Bacteroidetes* were more prone to developing allergic diseases after the age of 2 (Songjinda et al., 2007). *Bacteroidetes* and *Firmicutes* are the most common phyla in fecal microbiota, and they play important roles in gut health and immune regulation through the production of short-chain fatty acids (SCFAs) (Brody, 2020). In general, the presence of *Bacteroidetes* was often linked to a higher production of acetate and propionate, whereas the presence of *Firmicutes* was often linked to a higher production of butyrate (den Besten et al., 2013). Of all the SCFAs, butyrate is a key component in keeping the colon healthy by serving as a source of energy for colonic epithelial cells and regulating tight junction expression to preserve the integrity of the gut barrier (Rauf et al., 2022). Lack of butyrate can cause transfer of inflammatory molecules and antigens to the submucosa and the circulatory system, leading to localized and systemic inflammatory reactions. The prevalence of *Bacteroidetes* over *Firmicutes* may decrease the production of butyrate, thereby impacting the integrity of the gut barrier (Rauf et al., 2022) and the gut permeability of allergic disease patients is significantly higher than that of healthy controls (Benard et al., 1996; Majamaa and Isolauri, 1996). This implies that alterations in the abundance of *Bacteroidetes* and *Firmicutes* have an impact on the incidence and progression of allergic diseases, prompting the need for more research to clarify the connection between the level of butyrate-producing bacteria and intestinal permeability in patients with atopic diseases.

At the family level, there was a significant increase in the relative abundance of *Enterobacteriaceae* in the AR group. *Enterobacteriaceae* are Gram-negative facultative anaerobes, including intestinal bacteria such as *Escherichia coli*, *Shigella*, and *Salmonella*. Some strains of *Enterobacteriaceae* can adhere to the intestinal mucosa and stimulate immune reactions (Kim et al., 2018). Studies have found that in patients with allergies, the quantity of *Enterobacteriaceae* increases significantly. This phenomenon could be associated with the disturbance of the gut microbiota, leading to potential impacts on host immune and allergic responses (Zimmermann et al., 2019). Because *E. coli* and *Shigella* have the same 16S rRNA gene sequence, it is difficult to identify the specific adherent *Enterobacteriaceae* species only by 16S sequencing. Therefore, we will perform metagenomic sequencing in the future to determine the exact types of adherent *Enterobacteriaceae*.

In the AR group, the relative abundance of *Subdoligranulum*, *Agathobacter*, and *Roseburia* at the genus level was significantly lower when compared to healthy controls. *Subdoligranulum*, a member of the *Ruminococcaceae* family, can activate the neonatal Treg cells’ MyD88/ROR-γt pathway, thus effectively preventing food allergies (Duncan et al., 2004). The newly discovered *Agathobacter* belongs to the *Lachnospiraceae* family (Ahn et al., 2016) and *Roseburia* has become a potential health marker for gut microbiota due to its anti-inflammatory properties (Tamanai-Shacoori et al., 2017). *Subdoligranulum*, *Agathobacter*, and *Roseburia* can all produce butyrate, promoting gut health by providing energy to host cells and maintaining the integrity of the gut barrier (Ahn et al., 2016; Deaver et al., 2018). The decrease in relative abundance of *Subdoligranulum*, *Agathobacter*, and *Roseburia* in children with AR may indicate a decrease in butyrate levels and gut barrier disruption.

The cellular mechanism behind AR primarily involves the differentiation of CD4^+^ T cells into Th2 cells. This results in an imbalance of the Th1/Th2 ratio, thus promoting B cell activation and class switching to IgE (Kulkarni et al., 2011). Additionally, it causes the differentiation of B cells into IgE-producing plasma cells, as well as mast cell and eosinophil activation to release inflammatory mediators (Wang et al., 1992; Iinuma et al., 2018). Previous studies have demonstrated that an imbalanced gut microbiota can activate innate and adaptive immune cells, which may contribute to the onset of atopic diseases in children (Fujimura et al., 2016). In addition, SCFAs, which are metabolic products of gut microbiota, serve as mediators of communication between the immune system and gut microbiota (Milligan et al., 2017). SCFAs facilitate the differentiation of T cells into both regulatory and effector T cells, maintaining the balance between anti-inflammatory/pro-inflammatory responses, and promoting the establishment of immune tolerance (Park et al., 2015). Our study discovered alterations of gut microbiota in AR children, particularly, a reduction in the prevalence of butyrate-producing bacteria. This finding possibly supports the conclusion that SCFAs can prevent allergic respiratory diseases through their effects on T cells (Cait et al., 2018).

This study has some limitations. First, the sample size is relatively small, as most of the children had already used corticosteroids or antihistamines before their visit to the hospital. Second, the classification level in this study is relatively high, mainly at the phylum, family, and genus levels. Therefore, further research is needed to explore the changes in gut microbiota at the species level in children with AR to provide more precise microbiota-based treatments in the future. Lastly, as the role of gut microbiota metabolites in diseases becomes increasingly important, future studies will also perform metabolomics to further understand the changes in gut microbiota metabolites in children with AR.

## 5. Conclusion

In summary, we observed a close relationship between childhood AR and gut microbiota, confirming the changes in gut microbiota composition and diversity in children with AR. We believe that gut microbiota has the potential for diagnosis and treatment of childhood AR, and also provides some ideas for mechanism of AR.

## Data availability statement

The datasets presented in this study can be found in online repositories. The names of the repository/repositories and accession number(s) can be found at: https://figshare.com/s/8b5ae4eaa3489e55578c.

## Ethics statement

The experimental procedures were approved by the Ethics Committee of Xijing Hospital at the Fourth Military Medical University in Xi’an, China (approval number: KY20222169-F-1). Written informed consent to participate in this study was provided by the participants’ legal guardian/next of kin.

## Author contributions

PZ, XZ, HT, FJ, ZJ, HW, YZ, JL, JZ, and XS contributed to the study conception and design. PZ, XZ, and HT had the idea for the article. HW, YZ, and JL performed the data collection. The data analysis was performed by PZ and XZ. The first draft of the manuscript was written by PZ. XS and JZ critically revised the work. FJ and ZJ commented on several drafts. All authors contributed to the article and approved the submitted version.

## Funding

This work was supported by the National Natural Science Foundation of China (82170026); Technological Innovation Guidance Special Project of Shaanxi Province (2022QFY01-09); Natural Science Basic Research Plan in Shaanxi Province (2022JQ-764) and Discipline Promotion Project of Xijing Hospital (XJZT18MJ23 and XJZT21L11); and Clinical Research Project of Air Force Military Medical University (2022LC2210).

## Conflict of interest

The authors declare that the research was conducted in the absence of any commercial or financial relationships that could be construed as a potential conflict of interest.

## Publisher’s note

All claims expressed in this article are solely those of the authors and do not necessarily represent those of their affiliated organizations, or those of the publisher, the editors and the reviewers. Any product that may be evaluated in this article, or claim that may be made by its manufacturer, is not guaranteed or endorsed by the publisher.
