# Supplementary material for: Microbial signature of intestine in children with allergic rhinitis
Source: Front Microbiol. 2023 Jul 25;14:1208816. doi: 10.3389/fmicb.2023.1208816 (PMC10408450; doi:10.3389/fmicb.2023.1208816)
Supplement: Supplementary file 1 [file Table_1.DOCX]

Supplementary Material

**Microbial signature of intestine in untreated children with allergic rhinitis**

Zhang Panpan ^1, †^ Zhou Xuehua ^1, †^ Tan Hong^1, †^ Jian Fangfang ^1^ Jing Zenghui ^1^ Wu Huajie ^1^ Zhang Yao ^1^  Luo Jianfeng ^1^  Zhang Juan ^1, *^  Sun Xin ^1, *^

^†^ These authors contributed equally to this work and share first authorship

^1^ Department of Pediatrics, Xijing Hospital, the Fourth Military Medical University, Xi’an, Shaanxi, China

*** Correspondence:**

Co-corresponding author：Zhang Juan

[E-mail](mailto:E-mail): [805124257@qq.com](mailto:805124257@qq.com)

Corresponding Author: Sun Xin

[E-mail](mailto:E-mail): sunxin6@fmmu.edu.cn

# Supplementary Tables

Supplement Table 1. Quality of life scores of allergic rhinitis children

| Quality of Life | No  (0 point） | Yes,slight  （1 point） | Yes,moderately（2 point） | Yes,greatly（3 point） | Yes,very greatly（4 point） |
| --- | --- | --- | --- | --- | --- |
| Reduced productivity at work/home |  |  |  |  |  |
| Poor mental concertration |  |  |  |  |  |
| Reduced thinking power |  |  |  |  |  |
| Impaired reading book/newspaper |  |  |  |  |  |
| Reduced memory loss |  |  |  |  |  |
| Limitation of outdoor life |  |  |  |  |  |
| Limitation of going out |  |  |  |  |  |
| Hesitation visiting friend or relatives |  |  |  |  |  |
| Reduced contact with friends or others by telephone or conversation |  |  |  |  |  |
| Not an easy person to be around |  |  |  |  |  |
| Impaired sleeping |  |  |  |  |  |
| Tiredness |  |  |  |  |  |
| Fatigue |  |  |  |  |  |
| Frustration |  |  |  |  |  |
| Imitability |  |  |  |  |  |
| Depression |  |  |  |  |  |
| Unhappiness |  |  |  |  |  |
