# Supplementary material for: Microbial signature of intestine in children with allergic rhinitis
Source: Front Microbiol. 2023 Jul 25;14:1208816. doi: 10.3389/fmicb.2023.1208816 (PMC10408450; doi:10.3389/fmicb.2023.1208816)
Supplement: Supplementary file 2 [file Table_2.DOCX]

**Supplement Table 2.** The total number of raw reads, reads after quality filtering and paired-end reads stitched together for each sample.

| SampleID | No. of raw reads seqs | No. of reads after quality filtering | No. of paired-end reads |
| --- | --- | --- | --- |
| AR01 | 106067 | 61446 | 121566 |
| AR02 | 94235 | 61446 | 123115 |
| AR03 | 104592 | 61446 | 116881 |
| AR04 | 92149 | 61446 | 116659 |
| AR05 | 91469 | 61446 | 114936 |
| AR06 | 92081 | 61446 | 120914 |
| AR07 | 100078 | 61446 | 120769 |
| AR08 | 93169 | 61446 | 115999 |
| AR09 | 102605 | 61446 | 118893 |
| AR10 | 89472 | 61446 | 118916 |
| AR11 | 97902 | 61446 | 120672 |
| AR12 | 94349 | 61446 | 122709 |
| AR13 | 104371 | 61446 | 125446 |
| AR14 | 103546 | 61446 | 120549 |
| AR15 | 98755 | 61446 | 117716 |
| AR16 | 107553 | 61446 | 123224 |
| AR17 | 109981 | 61446 | 124689 |
| AR18 | 89490 | 61446 | 116403 |
| AR19 | 85725 | 61446 | 117368 |
| AR20 | 105549 | 61446 | 119263 |
| AR21 | 102819 | 61446 | 119606 |
| AR22 | 101342 | 61446 | 117240 |
| AR23 | 98273 | 61446 | 116560 |
| AR24 | 65399 | 61446 | 85787 |
| HCs01 | 81468 | 61446 | 108305 |
| HCs02 | 91537 | 61446 | 117641 |
| HCs03 | 78711 | 61446 | 103432 |
| HCs04 | 98201 | 61446 | 117085 |
| HCs05 | 90159 | 61446 | 109425 |
| HCs06 | 95004 | 61446 | 115749 |
| HCs07 | 96338 | 61446 | 118636 |
| HCs08 | 90266 | 61446 | 103167 |
| HCs09 | 95239 | 61446 | 121583 |
| HCs10 | 96881 | 61446 | 119385 |
| HCs11 | 98398 | 61446 | 124214 |
| HCs12 | 69730 | 61446 | 99122 |
| HCs13 | 94316 | 61446 | 116105 |
| HCs14 | 83472 | 61446 | 119895 |
| HCs15 | 61446 | 61446 | 75432 |
| HCs16 | 93951 | 61446 | 118248 |
| HCs17 | 93068 | 61446 | 113963 |
| HCs18 | 97104 | 61446 | 115941 |
| HCs19 | 103577 | 61446 | 124721 |
| HCs20 | 67187 | 61446 | 95891 |
| HCs21 | 95461 | 61446 | 115813 |
| HCs22 | 96467 | 61446 | 125074 |
| HCs23 | 93548 | 61446 | 119880 |
| HCs24 | 94546 | 61446 | 113909 |
| HCs25 | 86759 | 61446 | 121948 |

AR= allergic rhinitis group; HCs = healthy control group.
