# Supplementary material for: Microbial signature of intestine in children with allergic rhinitis
Source: Front Microbiol. 2023 Jul 25;14:1208816. doi: 10.3389/fmicb.2023.1208816 (PMC10408450; doi:10.3389/fmicb.2023.1208816)
Supplement: Supplementary file 3 [file Table_3.DOCX]

**Supplement Table 3:Alpha-Diversity at all levels.**

|  | AR | HCs | *P* value |
| --- | --- | --- | --- |
| Chao1 index at the phylum level | 3.73±1.06 | 3.66±0.92 | 0.809 |
| Chao1 index at the family level | 8.60±5.25 | 9.66±4.60 | 0.453 |
| Chao1 index at the genus level | 26.51±17.70 | 25.80±14.75 | 0.880 |
| Shannon index at the phylum level | 0.79±0.18 | 0.87±0.16 | 0.112 |
| Shannon index at the family level | 1.19±0.31 | 1.37±0.26 | 0.031 |
| Shannon index at the genus level | 1.52±0.46 | 1.81±0.39 | 0.025 |
| Simpson index at the phylum level | 0.52±0.11 | 0.47±0.59 | 0.058 |
| Simpson index at the family level | 0.41±0.15 | 0.34±0.84 | 0.033 |
| Simpson index at the genus level | 0.38±0.17 | 0.29±0.11 | 0.030 |

Student t-test was used for inter-group comparison.

AR= allergic rhinitis group; HCs = healthy control group.
