# Supplementary material for: Microbial signature of intestine in children with allergic rhinitis
Source: Front Microbiol. 2023 Jul 25;14:1208816. doi: 10.3389/fmicb.2023.1208816 (PMC10408450; doi:10.3389/fmicb.2023.1208816)
Supplement: Supplementary file 4 [file Table_4.DOCX]

**Supplement Table 4: Differential bacteria at the level of phylum, family, and genus**

|  | | AR | HCs | *P* value |
| --- | --- | --- | --- | --- |
| at the phylum | Bacteroidetes | 0.572±0.169 | 0.482±0.135 | 0.045* |
| at the family | Prevotellaceae | 0.002(0.002,0.003) | 0.002(0.001,0.002) | 0.011^#^ |
|  | Burkholderiaceae | 0.010(0.001,0.027) | 0.027(0.010,0.040) | 0.032^#^ |
|  | Enterobacteriaceae | 0.002(0.001,0.009) | 0.001(0.000,0.002) | 0.015^#^ |
| at the genus | Agathobacter | 0.007(0.004,0.031) | 0.035(0.018,0.082) | 0.001^#^ |
|  | Parasutterella | 0.002(0.000,0.014) | 0.013(0.002,0.028) | 0.002^#^ |
|  | Roseburia | 0.004(0.001,0.008) | 0.011(0.005,0.025) | 0.002^#^ |
|  | Subdoligranulum | 0.002(0.001,0.007) | 0.012(0.004,0.020) | 0.002^#^ |

*=Student t-test was used for inter-group comparison;

^#^=Wilcoxon signed-rank test was used for inter-group comparison.

AR= allergic rhinitis group; HCs = healthy control group.
